# Bone transport for the management of severely comminuted fractures without bone loss

**DOI:** 10.1007/s11751-016-0241-y

**Published:** 2016-02-01

**Authors:** Mootaz F. Thakeb, Mahmoud A. Mahran, El-Hussein M. El-Motassem

**Affiliations:** The Department of Orthopedic Surgery, Ain Shams University, Cairo, Egypt

**Keywords:** Bone transport, Comminuted fractures, Ilizarov technique, Contained bone defect, Internal bone loss

## Abstract

This study aims to provide a new method for treatment of severely comminuted fractures without bone loss using the well-known technique of bone transport. Sixteen patients suffering from severely comminuted fractures with closed soft tissue injury were prospectively treated using bone transport by Ilizarov circular fixator. There were 14 male and 2 female patients. The mean age was 36.5 years (27–45). There were 13 proximal tibial metaphyseal fractures, one tibial diaphyseal fracture and two femoral distal metaphyseal fractures. All patients had closed soft tissue. The mean length of the comminution gap was 50.3 mm (40–64). Fracture healing occurred in 15 patients. The mean healing time was 23.4 weeks (14–30). No bone stimulating procedures were needed for either the fracture or distraction site. Using the IOWA knee and ankle score for assessment of the 15 patients who completed treatment: the functional outcome for the knee was excellent in 11 patients, good in three and fair in one. The ankle score was excellent in 12 patients, good in two and fair in one. According to Paley and Maar’s, bone results were excellent in 14 patients, good in one patient and poor in the patient who had failure of the procedure. The results achieved in this work are encouraging to keep on applying this technique to treat fractures that meet the following criteria: metaphyseal, with total circumferential comminution involving more than 4 cm of the bone length.

## Introduction

Bone transport for the management of traumatic bone loss is a well-known technique [1–4]. Bone loss may occur from extrusion of fragments at the time of injury or during debridement of an open fracture when devitalized segments of bone are removed. This creates a segmental defect or gap between the remaining bone ends.

A severely comminuted fracture with intact soft tissue envelope, having circumferentially widely separated fragments that involve more than 2 cm of the bone length, should be considered as a fracture with “contained defect” or “internal bone loss.” Open fractures with segmental defects more than 2 cm are unlikely to heal spontaneously following bone stabilization alone. Fractures with 50 % or more of circumferential bone loss require bone graft to restore normal volume and strength [5–8].

Comminuted fractures with contained bone defects present difficulties in management because of the high potential of reduced fragments’ viability, soft tissue compromise and problems with stabilization. Various surgical methods have been proposed for treating such complex fractures including: internal fixation by plates and screws, intramedullary nailing and external fixation [5, 9, 10].

Circular external fixator provides multilevel stabilization of the fractured limb segments with minimal disruption of the soft tissue envelope. It is particularly useful where bone gaps need reconstruction by distraction osteogenesis.

This is a report of using bone transport technique to manage closed comminuted fractures with “contained bone defects” to bridge the gap and achieve healing in a reasonable time.

## Patients and methods

Between June 2005 and January 2011, 16 patients suffering from severely comminuted fractures with closed soft tissue injury were treated using bone transport by Ilizarov circular fixator.

There were 14 males and 2 females. The mean age was 36.5 years (range 27–45). There were 14 tibial fractures (13 proximal metaphyseal and one diaphyseal), and two femoral distal metaphyseal fractures. According to AO/OTA system [11], ten fractures were classified as 41.A3, three as 41.C2, one as 42.C3, one as 33.A3.3 and one fracture as 33.C2.3.

All patients had closed soft tissue injuries that were graded according to Tscherene and Gotsen method [12]. Eight were grade I, five were grade II and three were grade III. Nine patients had their fractures as isolated injury while seven had other fractures (Table 1). The mechanism of injury was road traffic accident in 11 patients, trauma from a heavy object in three patients and falls from a height in two patients.

**Table 1:** Patients data

Case	Age	Gender	Grade of soft tissue injury	AO classification	Comminution gap (mm)	Associated injuries	Primary treatment	Time to frame (days)
1	27	M	2	41-A3	64	Contralateral fracture distal humerus	Back Slab	7
2	45	M	1	41-A3	52	–	Back Slab	1
3	36	M	3	41-C2	58	–	Temporary fixator	28
4	40	M	1	33-C2.3	45	Ipsilateral fracture distal radius	Back Slab	1
5	38	M	2	41-A3	42	–	Back Slab	10
6	28	M	1	33-A3.3	40	Contralateral fracture tibia	Back Slab	1
7	32	F	1	41-A3	55	–	Back Slab	1
8	43	M	1	41-A3	52	–	Back Slab	1
9	35	M	2	42-C3	40	–	Back Slab	7
10	33	M	3	41-A3	60	Contralateral fracture Posterior wall acetabulum	Temporary fixator	25
11	44	M	2	41-C2	50	–	Temporary fixator	18
12	28	M	1	41-A3	40	Contralateral fracture femur	Back Slab	1
13	34	F	1	41-A3	44	–	Back Slab	1
14	36	M	3	41-C2	50	–	Temporary fixator	28
15	45	M	2	41-A3	58	Ipsilateral fracture humerus	Back Slab	10
16	40	M	1	41-A3	55	Ipsilateral fracture calcaneus	Back Slab	1

**Fig. 1:**
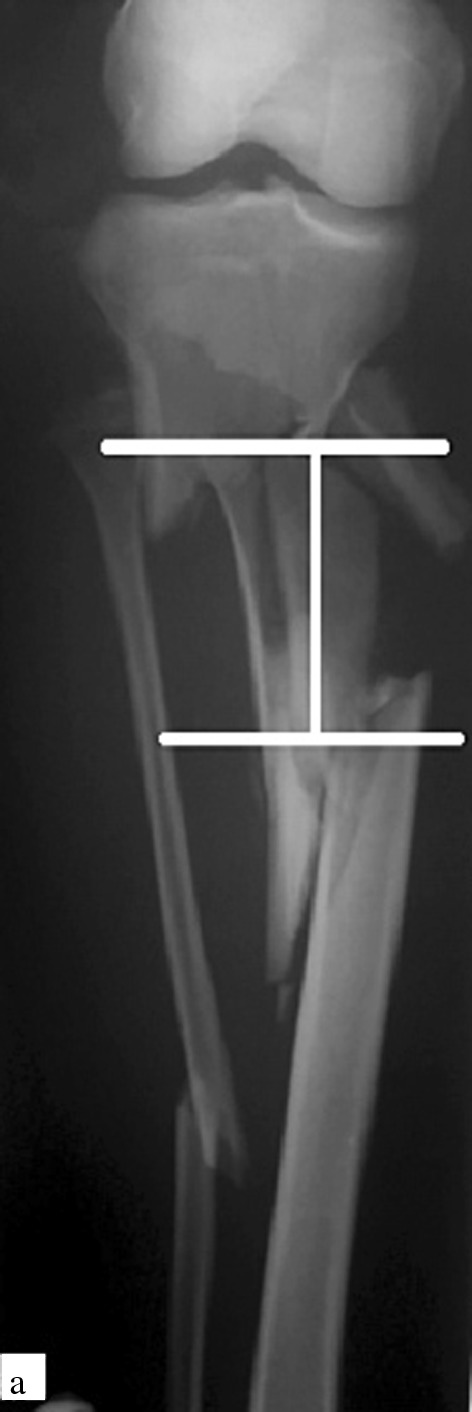
Antero-posterior radiograph showing comminuted fracture proximal tibia, 41-A3, with a segmental comminution gap of 64 mm

The length of the segmental comminution gap was measured between two points of circumferentially intact bone at the proximal and distal main bone segments (Fig. 1). The mean length of this gap was 50.3 mm (range 40–64).

Four patients had their fracture initially immobilized with a mono-lateral spanning external fixator. Twelve patients had their fractures immobilized in plaster back slab until definitive stabilization. The mean time to definitive surgery was 8.8 days (range 1–28). The operative delay in some patients was due to late referral from primary centers.

Four patients (three type 41-C2 proximal tibia and one type 33-C2.3 distal femur) had their articular fragments reduced and fixed percutaneously with one or two cannulated 6.5 screws and washers. Tibial fractures were stabilized with an Ilizarov frame; each segment of bone was fixed either by two rings or a single ring with a drop wire or half pin on either side. Each ring was fixed by at least two tensioned 1.8-mm wires and a 6-mm predrilled half pin. The femoral frames comprised two femoral arches proximally and two distal rings. Three or four 6-mm half pins fixed each arch. Two tensioned wires and half pins were fixed to the rings.

The two femoral fractures and the tibial diaphyseal fracture had proximal to distal bone transport. The 13 proximal tibial fractures had distal to proximal bone transport. Corticotomies were done using predrilling technique.

**Fig. 2:**
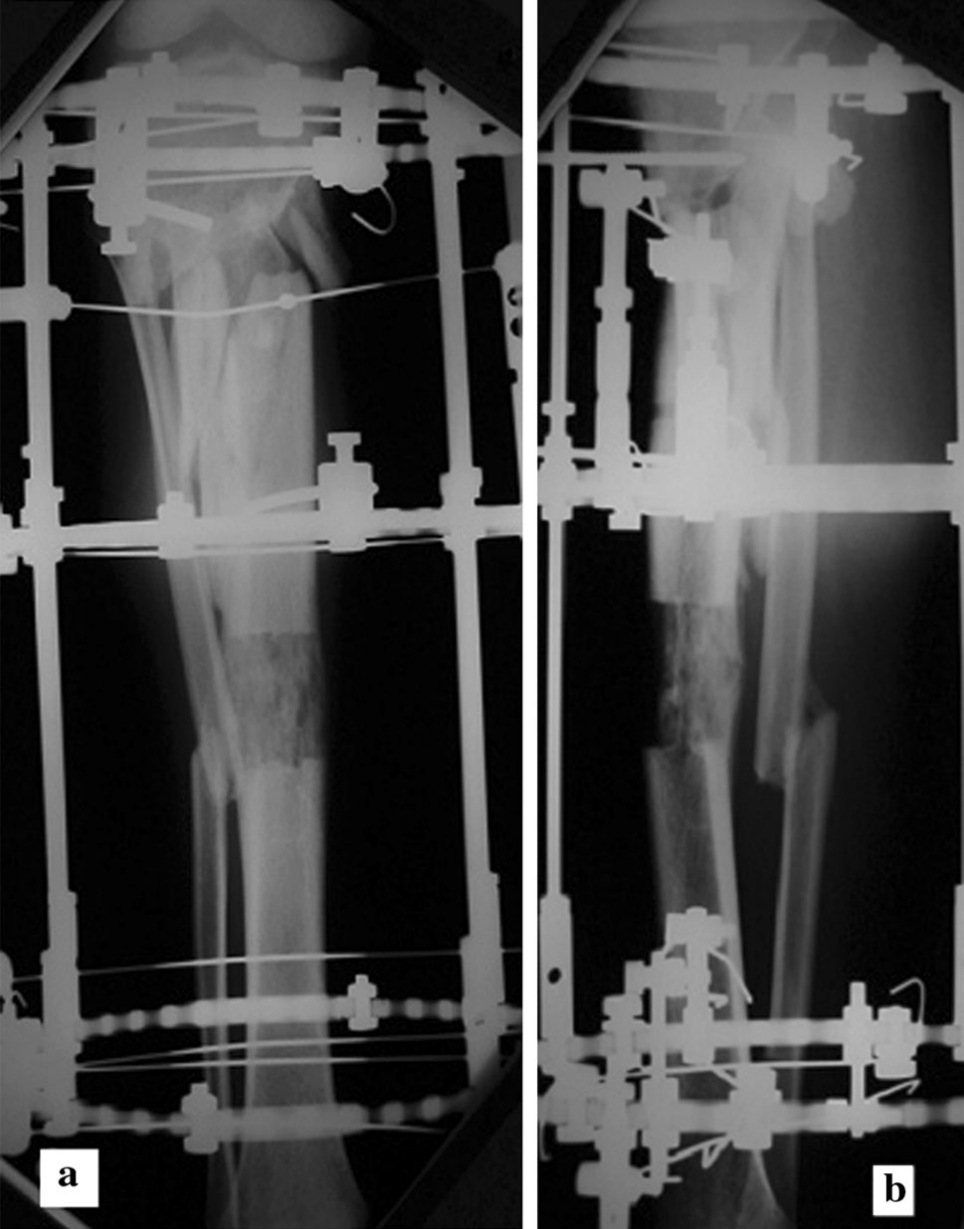
**a**, **b** Antero-posterior and lateral radiographs showing the distracted segment bridging the comminution gap, with good consolidation

Postoperatively, patients were allowed partial weight bearing unless contraindicated by the presence of other injuries. Knee and ankle range of motion exercises started on the first postoperative day or as tolerated by the patient. Distraction started after a latent period of 7 days at a rate of 0.25 mm/6 h. This continued till the comminuted fragments resisted transport. Thus, the amount of distraction was not necessarily equal to the preoperative measured length of contained defect (Fig. 2).

Pin site care included daily removal of crusts with normal saline and application of compressive dressing. Alcoholic chlorhexidine antiseptic solution was used only if pin site inflammation occurs.

Patients were followed up weekly during the distraction period and then every 2 weeks till frame removal. All frames were dynamized 2 weeks before removal.

Healing was determined radiologically and clinically: radiologically, callus bridging the fracture site and the appearance of three cortices bridging the distraction corticotomy on antero-posterior and lateral radiographs indicated healing. Clinical fracture healing was determined when the patient was able to bear weight freely without supporting aids after frame dynamization.

The final follow-up was done after 12 months using the IOWA [13] knee and ankle functional score and Paley and Maar’s bone results [4].

## Results

**Fig. 3:**
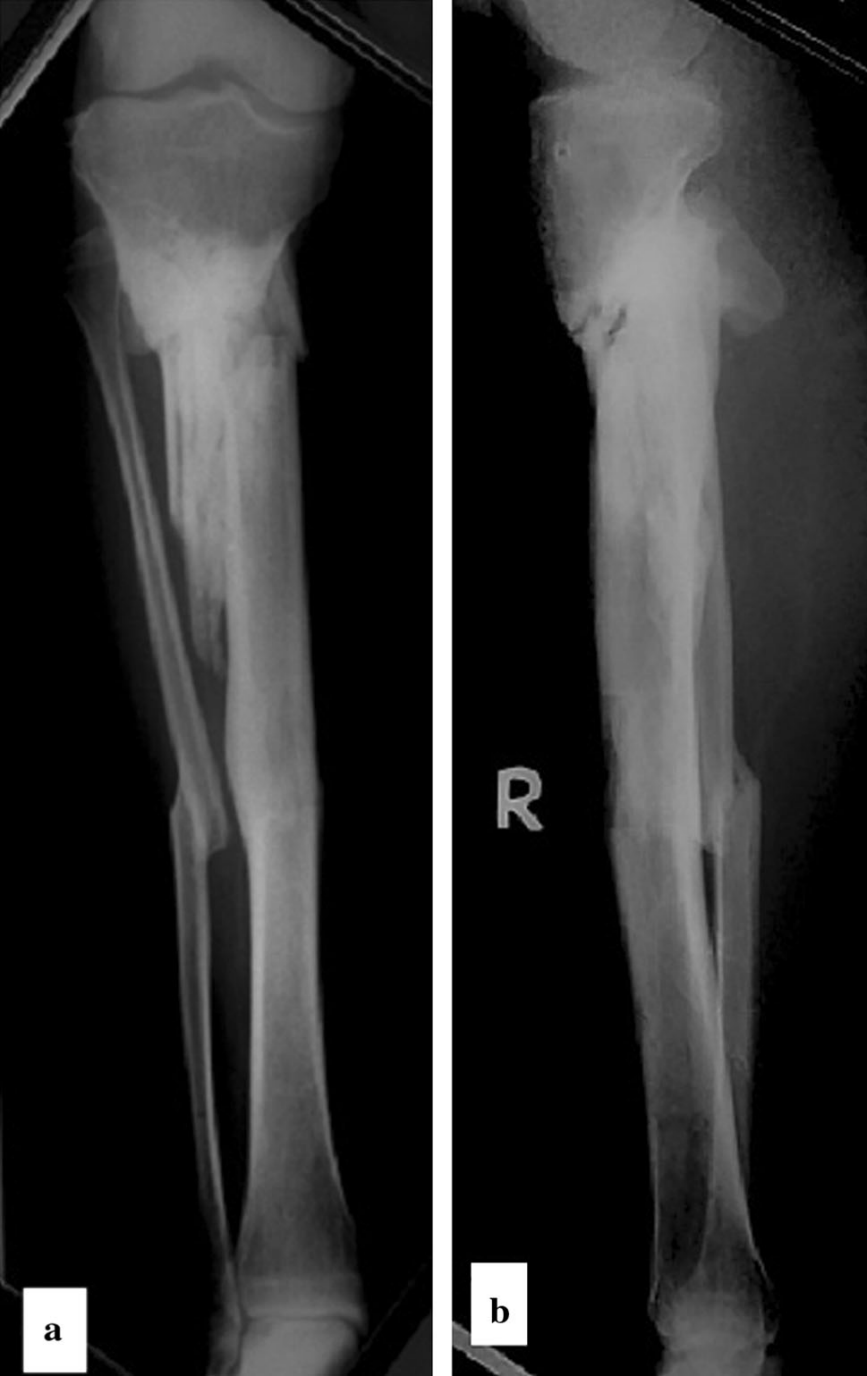
**a**, **b** Antero-posterior and lateral radiographs at final follow-up showing fully consolidated fracture and distraction sites with 10 mm medial translation of the mechanical axis

Fifteen fractures healed uneventfully (Fig. 3). The mean healing time was 23.4 weeks (range 14–30). The mean consolidation time for the distraction site was 19 weeks (range 10–24). No bone healing stimulating procedures were needed for either the fracture or distraction site (Table 2). The mean length gained (the transported distance) was 39.4 (range 20–50) mm.

**Table 2:** Results

Case	Time to healing		Amount of distraction (mm)	Comminution gap (mm)	Residual deformity	IOWA score		Bone results
	Distraction site (weeks)	Fracture site (weeks)				Knee	Ankle	
1	22	24	45	64	Medial translation 10 mm	96	94	Excellent
2	20	22	42	52	–	98	100	Excellent
3	24	24	45	58	5° Varus, 1 cm shortening	90	96	Excellent
4	15	20	35	45	–	86	100	Excellent
5	12	16	30	42	1 cm shortening	94	98	Excellent
6	10	15	25	40	–	90	96	Excellent
7	24	28	50	55	5° Varus, 1 cm shortening	96	100	Excellent
8	20	26	40	52	–	100	100	Excellent
9	–	–	20	40	–	–	–	Poor
10	22	28	50	60	1.5 cm shortening	84	79	Excellent
11	18	24	44	50	–	92	94	Excellent
12	12	14	25	40	5° Varus	94	98	Excellent
13	18	24	40	44	10° varus	89	92	Good
14	22	28	42	50	1.5 cm shortening	74	89	Excellent
15	24	30	50	58	5° Valgus	98	100	Excellent
16	22	28	48	55	1 cm shortening	92	83	Excellent

Pin site infection was the most common problem encountered in almost all patients and treated with oral antibiotics. None progressed to deep infection or required exchange of wires or half pins.

One patient (with 42-C3 diaphyseal fracture) was not able to tolerate the procedure; the frame was removed prematurely. A back slab was applied temporarily for 4 weeks until consolidation of the distraction gap. Revision of fixation was performed with an unreamed interlocking nail and iliac crest bone graft used. This patient was excluded from the final functional assessment and was rated as a poor bony result due to failure of the bone transport procedure alone to achieve healing.

The IOWA knee and ankle score was used for assessment of the 15 patients who completed treatment. The functional outcome for the knee was excellent in 11 patients, good in three and fair in one. The ankle score was excellent in 12 patients, good in two and fair in one. The patient who did not complete treatment with bone transport was not included in this final functional assessment and was considered as a failure of the procedure.

According to Paley and Maar’s scoring system, bone results for all 16 patients were excellent in 14 patients, good in one patient and poor in the patient who had failure of the procedure.

## Discussion

High-energy fractures have soft tissue compromise and a potentially reduced viability of bone ends which can alter the normal healing process with considerable delay in union expected. The method chosen for treatment of these fractures has a substantial effect on the local mechanical and biological environment. The treatment strategy for these fractures focuses on fixation stability while respecting the biological reserve.

Restoration of limb length and alignment together with preservation of function are the main goals in treatment of these injuries. Severely comminuted metaphyseal fractures challenge the ability of standard implants to provide adequate stability. Fixation by plates and screws pose an additional surgical injury to an already compromised soft tissue envelope, even with minimally invasive plate designs. Furthermore, the presence of a segmental gap or defect will compromise the stability of plate fixation; when prolonged bone healing time is expected, failure may occur by cantilever loading [5, 14].

The use of intramedullary nails in comminuted fractures with contained metaphyseal defects is not suitable. Short metaphyseal proximal tibial or distal femoral segments are difficult to control and malalignment can be difficult to avoid, even with the use of nails with multidirectional locking screws [5, 15].

The Ilizarov external fixator can be applied with minimal soft tissue disruption. Moreover, it offers the mechanical advantage of resisting all prevailing loads except the axial ones that are beneficial for osteogenesis. The multidirectional fixation can adequately stabilize short metaphyseal segments allowing early weight bearing and rehabilitation; additionally, it has the versatility for correcting any residual postreduction deformities.

A severely comminuted fracture forms a “contained defect” comprised of fracture fragments with reduced viability. In this series, the bone transport technique was used knowing corticotomy increases blood flow to the limb [16] and the transported bone segment is able to bridge the area of the contained defect and avoid need for bone graft.

Pin site infections encountered in all patients were successfully controlled with oral antibiotics. This is considered a problem of external fixation used in limb reconstruction as opposed to an obstacle or true complication as described by Paley [17]. The only true complication was the non-union encountered with one patient with the diaphyseal fracture who did not tolerate the procedure.

The functional outcome in this series indicates the limited knee and ankle range of motion that can be encountered in some patients treated by Ilizarov method is temporary and can be resolved after frame removal. Early postoperative knee and ankle range of motion exercises are imperative to avoid this problem.

The mean time to bone healing was 23.4 weeks which the authors consider as a reasonable time for such fractures. This is comparable to the healing time reported for complex and segmental fractures stabilized by circular fixators [9, 10, 18].

## Conclusion

We describe a technique of bone transport to stimulate and overcome the comminution in high-energy fractures of the tibia and femur using the Ilizarov fixator as an alternative to other methods of stabilization with or without bone graft. The results presented confirm the suitability of the strategy for this cohort of patients who have metaphyseal fractures with total circumferential comminution involving more than 4 cm of the bone length.
